# Compression Behavior and Vibrational Properties of New Energetic Material LLM-105 Analyzed Using the Dispersion-Corrected Density Functional Theory

**DOI:** 10.3390/molecules26226831

**Published:** 2021-11-12

**Authors:** Tianming Li, Junyu Fan, Zhuoran Wang, Hanhan Qi, Yan Su, Jijun Zhao

**Affiliations:** 1 Department of Physics, Taiyuan Normal University, Jinzhong 030619, China; 2 Key Laboratory of Materials Modification by Laser, Ion and Electron Beams (Dalian University of Technology), Ministry of Education, Dalian 116024, China; litianming21@mails.ucas.ac.cn (T.L.); wangzr@ihep.ac.cn (Z.W.); qhh0818@mail.ustc.edu.cn (H.Q.); zhaojj@dlut.edu.cn (J.Z.); 3 Institute of Computational and Applied Physics, Taiyuan Normal University, Jinzhong 030619, China

**Keywords:** high pressure, vibrational properties, energetic material, anisotropy, uniaxial compression

## Abstract

The 2,6-diamino-3,5-dinitropyrazine-1-oxide (LLM-105) is a newly energetic material with an excellent performance and low sensitivity and has attracted considerable attention. On the basis of the dispersion-corrected density functional theory (DFT-D), the high-pressure responses of vibrational properties, in conjunction with structural properties, are used to understand its intermolecular interactions and anisotropic properties under hydrostatic and uniaxial compressions. At ambient and pressure conditions, the DFT-D scheme could reasonably describe the structural parameters of LLM-105. The hydrogen bond network, resembling a parallelogram shape, links two adjacent molecules and contributes to the structure stability under hydrostatic compression. The anisotropy of LLM-105 is pronounced, especially for Raman spectra under uniaxial compression. Specifically, the red-shifts of modes are obtained for [100] and [010] compressions, which are caused by the pressure-induced enhance of the strength of the hydrogen bonds. Importantly, coupling modes and discontinuous Raman shifts are observed along [010] and [001] compressions, which are related to the intramolecular vibrational redistribution and possible structural transformations under uniaxial compressions. Overall, the detailed knowledge of the high-pressure responses of LLM-105 is established from the atomistic level. Uniaxial compression responses provide useful insights for realistic shock conditions.

## 1. Introduction

Energetic materials (EMs), such as explosives, propellants, and pyrotechnics, are widely used for civilian and military purposes [1,2]. EMs can undergo multiple phase transitions during detonation, in which the molecules are modified and rearranged under high-temperature and high-pressure conditions. Furthermore, the evolutions of the pressure-dependent physical and chemical properties of EMs are important in the control of their sensitivity, performance, and safety [3,4]. Recently, 2,6-diamino-3,5-dinitropyrazine-1-oxide (LLM-105), a new generation of high-energy and low-sensitivity EM, was synthesized by Lawrence Livermore Laboratories in the United States, and its foundational properties attracted attention.

Single-crystal X-ray diffraction experiment [5] showed that the LLM-105 crystal contains C, H, N, and O with a space group of *P*2_1_/*n* in the monoclinic crystal, in which every H atom is involved in intramolecular hydrogen-bonding interactions with its neighboring O atom and in intermolecular interactions with adjoining molecules [6]. At ambient conditions, LLM-105 with a wave-like π–π stacking arrangement possesses rich intra- and intermolecular hydrogen-bonded networks, which buffer the perturbations of the external environment on the LLM-105 crystal [7,8].

Several experimental studies showed that the LLM-105 crystal exhibits a strong mechanical and thermal stability under external temperature and pressure loading. X-ray diffraction, Raman spectroscopy, and infrared spectroscopy are powerful experimental tools to elucidate the changes in the crystalline structure under extreme conditions. Gump et al. [9]. and Stavrou et al. [10]. used X-ray diffraction technology to determine the isothermal equation of the state of unreacted LLM-105 crystal. Their results demonstrated that the phase structure of LLM-105 remained stable at a pressure up to 20 GPa and temperature up to 513 K. Recently, Zhang et al. [11]. confirmed that the strong intermolecular and intramolecular hydrogen bond network of LLM-105 crystal contributes to the stability of the structure through high-pressure Raman and infrared spectroscopy. Importantly, the first phase transition of LLM-105 is confirmed near the hydrostatic pressure of 30 GPa, in which several Raman peaks disappear, and vibrations show an abrupt change at about 30 GPa. In addition, by analyzing the photoluminescence and absorption spectra [12], the LLM-105 crystal has an adjustable indirect band gap and a phase change in the electronic structure around 10 GPa. 

Alternatively, theoretical calculations can give several important insights at atomic and molecular levels on the structural evolution and anisotropy properties of LLM-105 crystals under hydrostatic compression. Wu et al. [13]. used the standard density functional theory (DFT) to simulate the structural changes in the LLM-105 crystal at a hydrostatic pressure of 0–50 GPa. They pointed out that the evident irregular changes in lattice parameters, including unit–cell angles, bond lengths, bond angles, and band gaps, were closely related with the structural transformations of LLM-105. However, Stavrou et al. [10] conducted first-principles molecular dynamics simulations to verify that the ambient pressure phase of LLM-105 could remain stable up to 20 GPa. On the basis of the dispersion-corrected DFT calculations, Manaa et al. [14]. found no evidence for structural phase transitions of up to 45 GPa by calculating the equation of state (EOS) of the LLM-105 crystal under hydrostatic compression. Zong et al. [15]. used the generalized gradient approximation parameterized by Perdew–Burke–Ernzerhof (GGA–PBE method) to simulate the properties of LLM-105 at a pressure of 0–100 GPa and found that the structural phase change occurs at 35 GPa. In addition, the decomposition mechanism of LLM-105 is studied on the basis of DFT. Zhang et al. [16]. showed that the intramolecular hydrogen transfer is reversible during the decomposition process of LLM-105, and the reversible hydrogen transfer can buffer the external stimuli caused by energy transfer and slight structural changes. 

Although considerable experimental and theoretical efforts focused on the compression behaviors and thermal decomposition mechanism [17,18,19,20,21,22,23], the anisotropic vibrational property and structural modification of LLM-105 under different pressure loading conditions require further clarification. In this work, we performed systematic first-principles calculations to elucidate the crystalline structure and vibrational properties under hydrostatic and uniaxial compressions. Under high-pressure conditions, the hydrogen bond network between adjacent molecules maintained the stability of the crystal structure. The red-shift of vibrational modes was observed under hydrostatic and uniaxial compressions. The discontinuous change in high-wavenumber modes along a [001] orientation compression indicates the onset of possible structure transformations. The in-depth research in this area aids in the understanding of the structural response of the LLM-105 crystal under a high pressure, further providing theoretical support for understanding the stability of EMs.

## 2. Results and Discussion

### 2.1. Lattice Parameters of LLM-105 at Ambient and Pressure Conditions

Here, the lattice parameters of LLM-105 and the internal coordinates of the atoms are fully relaxed on the basis of DFT-D calculations at zero pressure. In Table 1, the calculated results are compared with previous experimental and theoretical data [5,9,10,13,15,18]. The DFT calculations with DFT-D2 schemes significantly improved the description of the crystal structure at ambient conditions. The calculated lattice parameters are in agreement with experimental values [5] and superior to previous first-principles calculations based on the force field [18] and parameterized CA-PZ and PW91 scheme [13], and close to the theoretical result by Zong et al. [15]. Specifically, the calculated lattice parameters are consistent with the experimental measurements. The relative errors of lattice parameters (including *a*, *b*, *c*, *V*, and *β*) are below 1.1%, indicating that the current PBE-D2 scheme gives a reasonable description of intermolecular interactions, including intermolecular hydrogen bonding.

**Figure 1:**
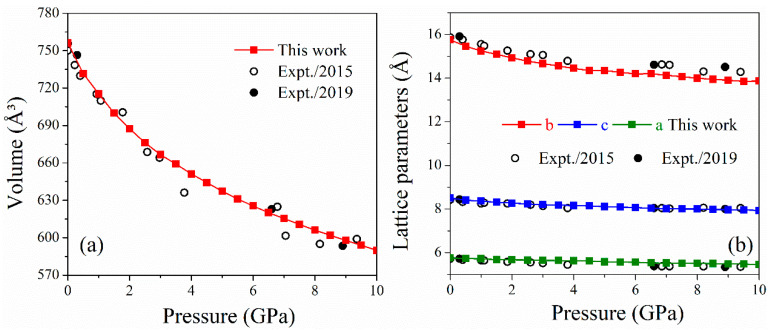
Variations in unit cell volume (**a**) and lattice parameters (**b**) under hydrostatic compression.

Figure 1 shows the evolutions of cell volume and lattice parameters of LLM-105 under pressure up to 10 GPa. Overall, the cell volume and lattice parameters of LLM-105 from the DFT-D calculations are reasonably consistent with experimental XRD data [10,11]. Figure 1a shows the variation in the cell volume of LLM-105 from our DFT-D calculations and previous experimental results as the pressure increases. Current DFT-D calculations reproduce the trend of pressure-induced volume compression from experiments, in which the volume compression at 10 GPa is up to 22%. The pressure-induced reductions in lattice parameters are displayed in Figure 1b. The lattice parameter *b* exhibits a significant reduction compared to lattice parameters *a* and *c* as the pressure increases, indicating that the LLM-105 crystal is easily compressible along the *b*-axis. This phenomenon occurs because the weak interlayer vdW interaction along the *b*-axis is more compressible than the relatively strong hydrogen bonds in the molecules along the *a-* and *c*-axes. The anisotropic compressibility is common feature for EMs [24,25] and reflects the strength of intermolecular interactions in different directions, which is related to the anisotropic molecular responses of LLM-105 under pressure loading.

In addition, third-order Birch–Murnaghan EOS are applied to fit the bulk modulus (*B*_0_) of LLM-105 to evaluate its stiffness on the basis of the calculated *P–V* relationship. The third-order Birch–Murnaghan EOS are given as follows [26]:

(1) $$P\left(V\right)=\frac{3B_{0}}{2}\left[{\left(\frac{V_{0}}{V}\right)}^{7/3}-{\left(\frac{V_{0}}{V}\right)}^{5/3}\right]\times \left\{1+\frac{3}{4}\left(B_{0}^{\prime }-4\right)\left[{\left(\frac{V_{0}}{V}\right)}^{2/3}-1\right]\right\}$$

where *P* represents the applied pressure, and *V*_0_ and *V* represent volumes at ambient and pressure conditions, respectively. *B*_0_ represents the bulk modulus and *B*_0_′ represents its first pressure derivative. 

Table 2 summarizes the bulk modulus (*B*_0_) and its derivative (*B*_0_′) of LLM-105, as well as previous experimental and theoretical results [9,10,11,14,15,18]. Our calculated *B*_0_ and *B*_0_′ are 16.03 and 8.67 GPa, respectively, which are in accordance with the reported experimental and theoretical data.

### 2.2. Raman Spectra of LLM-105 at Ambient and Hydrostatic Conditions

Raman spectroscopy is used to determine the crystal structure and chemical bonding of materials by identifying the vibrational modes of molecules in experimental and theoretical studies [27]. An atomistic level spectroscopic simulation can demonstrate experimental findings, thereby providing deep insights into structural modification and noncovalent interactions. For the space group *P*2_1_/n (2/m), a group theoretical analysis gives 228 vibrational modes (*Γ* = 57*A*_g_+ 57*B*_g_ + 57*A*_u_ + 57*B*_u_) for the LLM-105 crystal at ambient conditions. Among them, the calculated vibrations of even parity (g) are Raman-active. Thus, 57 Raman vibrations of each symmetry are present.

**Figure 2:**
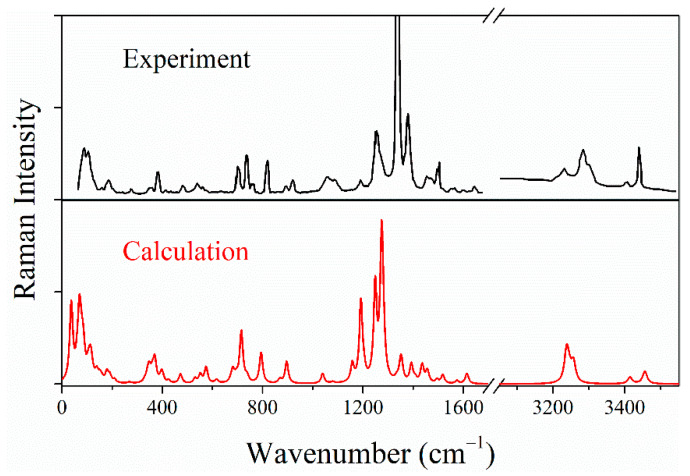
Calculated and experimental Raman spectrum of LLM-105 at ambient conditions.

In Figure 2, the calculated and experimental Raman spectra [11] of LLM-105 are present over a broad wavenumber range. The relative wavenumber and intensity of characteristic peaks are essentially consistent between calculated and experimental spectra. The high-wavenumber C–H and O–N–O stretch patterns have a larger deviation than other internal modes due to the lack of anharmonic effects and insufficient description of intermolecular interactions. In addition, the characteristics of the calculated and experimental internal/lattice vibrational modes, including wavenumber, assignment, and pressure-dependence, are presented in Table S1. Several coefficients of pressure dependence from theoretical calculations deviate from the experiments due to a lack of temperature effects under harmonic approximation.

**Figure 3:**
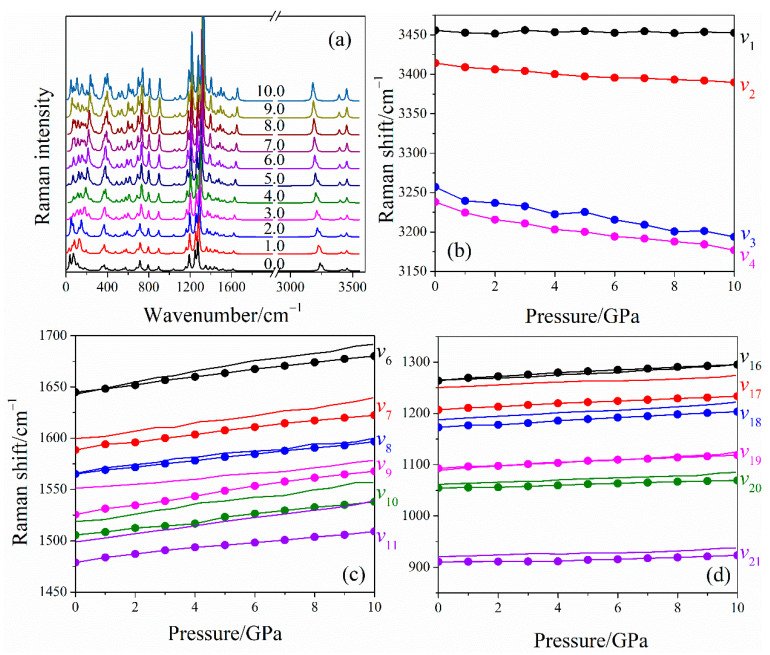
Evolutions of simulated Raman spectra (**a**) and wavenumber shifts (**b**–**d**) as a function of pressure under hydrostatic compression. Wavenumbers are shifted by the same amount to match the calculated and experimental values.

**Figure 4:**
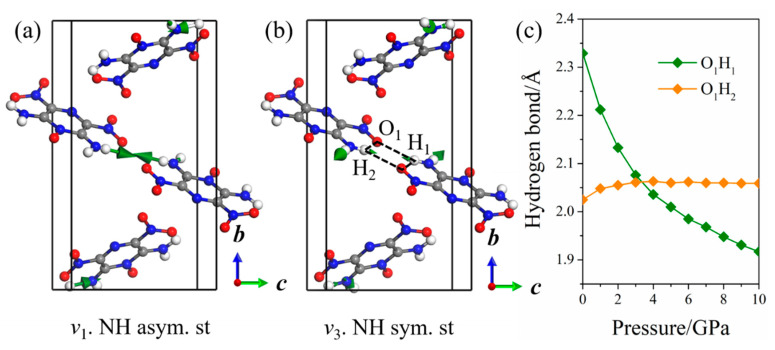
Schematic of high-frequency vibrational modes *v*_1_ (**a**) and *v*_3_ (**b**). Variation of select hydrogen bonds under hydrostatic pressure (**c**).

The pressure dependence of Raman spectra under hydrostatic compressions is shown in Figure 3a, and detailed comparisons of the selected Raman shift between calculated and experimental results are presented in Figure 3b–d. The Raman spectra in the experiment are concentrated in the middle- and low-wavenumber regions (<1700 cm^−1^). The trends of the calculated Raman shift of most modes are in agreement with the experimental measurements, which indicated that the current DFT-D scheme is relatively reliable. Additionally, all of these vibrational modes are blue-shifted due to the reduced intermolecular distance as a result of the enhanced intermolecular interactions under pressure. Importantly, the high-wavenumber modes (*v*_1_–*v*_4_) associated with the motion of the amino group are predicted at 3200−3500 cm^−1^. The asymmetric NH_2_ stretching (*v*_1_ and *v*_2_) has higher wavenumber than symmetric ones (*v*_3_ and *v*_4_). The NH_2_ stretching modes, including asymmetric *v*_2_, symmetric *v*_3_, and symmetric *v*_4_ vibrations, present a red-shift at 0–10 GPa, indicating that the LLM-105 crystal tends to be unstable with an elevated pressure. However, the wavenumber of the asymmetric *v*_1_ vibrational mode is nearly identical in the entire pressure range. From the vibrational pattern of LLM-105 in Figure 4a,b, modes *v*_1_ and *v*_3_ are from the motion of the amino group at site A. The N_1_–H_2_ bond participates in the *v*_1_ vibrational mode with a relatively strong contribution, whereas the N_1_–H_3_ bond participates in the *v*_3_ vibrational mode. In Figure 4c and Figure S1, the configuration of the hydrogen bond N_1_–H_2_···O_1_ is stable with an initial bond length and angle at 0–10 GPa; this is why the wavenumber of the *v*_1_ vibrational mode remains almost constant. The length of the N_1_–H_3_ bond does not change, whereas the bond angle of H_2_–N_1_–H_3_ continues to decrease, which can cause the red-shift of the *v*_3_ vibration mode. In addition, the hydrogen bond network between the adjacent molecules always maintains a parallelogram-like configuration (O_1_···H_1_···O_2_···H_2_, Figure 4b,c. When the pressure increases, the O···H-bonded molecular pairs, via nearby molecules and the cross-stacking of molecular layers for LLM-105, seem to be tightly confined, in which such special configurations can contribute to maintain the stability of the crystal structure under high-pressure conditions.

Table S1 shows that most vibrational modes of LLM-105 are combinations of cation and anion vibrations at ambient conditions. The Raman frequencies of these modes increase with the elevated pressure in most cases below 1600 cm^−1^. The highest coefficient (8.13 cm^−1^·GPa^−1^) is observed for the low-frequency internal mode *v*_29_, whereas lattice modes have coefficients less than 2.99 cm^−1^·GPa^−1^.

### 2.3. Raman Spectra of LLM-105 under Uniaxial Compressions 

#### 2.3.1. Stress Tensor and Shear Stress 

The high-pressure behavior of EMs under uniaxial compressions can be associated with their anisotropic shock sensitivity. The stress tensor and its derived shear stress are calculated to evaluate the anisotropic response under uniaxial loading and to quantify this sensitivity. On the basis of the calculated diagonal elements of the stress tensor, the principal stresses are obtained under uniaxial compressions in Figure S2. Three principal stresses, *σ*_xx_, *σ*_yy_, and *σ*_zz_, along the [100] and [010] orientations appear to increase under the uniaxial compression ratio (*V/V*_0_). However, the difference in variation trends and amplitudes clearly indicates the significant anisotropic behavior of LLM-105. For example, the largest principal stress *σ*_zz_ along the [001] orientation is 17.06 GPa at *V/V*_0_ = 0.78, which is larger than the *σ*_xx_ (12.07 GPa) along the [100] orientation and the *σ*_yy_ (11.94 GPa) along the [010] orientation. Thus, we suggest that the [001] orientation can be more sensitive than the [100] and [010] orientations. Furthermore, the average principal stress $\bar{\sigma }$, defined as $\bar{\sigma }=\left(\sigma_{xx}+\sigma_{yy}+\sigma_{zz}\right)/3$, is calculated as the function of *V/V*_0_ in Figure S3, which demonstrates the anisotropic behavior of LLM-105 under compression.

**Figure 5:**
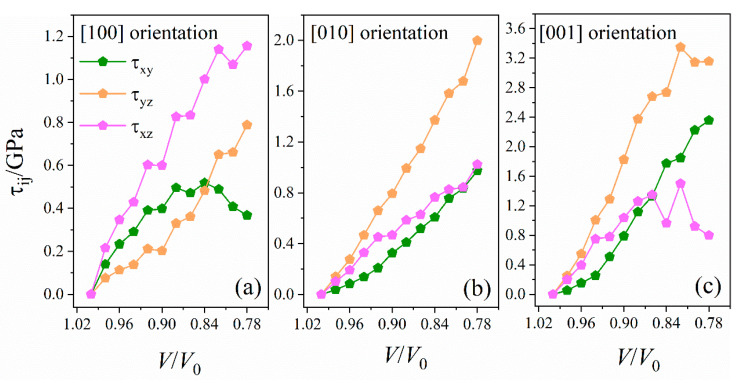
Shear stresses, *τ*_xy_, *τ*_xz_, and *τ*_yz_, as a function of compression ratio (*V/V*_0_) along [100] (**a**), [010] (**b**), and [001] (**c**) orientations.

Shear stresses, which are defined as$\;\tau_{xy}=\left(\sigma_{xx}-\sigma_{yy}\right)/2$, $\tau_{xz}=\left(\sigma_{xx}-\sigma_{zz}\right)/2$ and $\tau_{yz}=\left(\sigma_{yy}-\sigma_{zz}\right)/2$, are the major parameters used to calculate the mechanical anisotropy. The calculated shear stresses as a function of *V/V*_0_ under two uniaxial compressions are displayed in Figure 5. The shear stresses along the [010] orientation increase almost linearly, whereas those along the [100] and [001] orientations show a significant nonmonotonic behavior. Among them, the largest shear stresses along the [100], [010], and [001] orientations are 1.16 (*τ*_xz_), 2.00 (*τ*_yz_), and 3.35 (*τ*_yz_) GPa, respectively. These results demonstrate the anisotropic response of LLM-105 under uniaxial compressions, and shear stresses are the major parameters used to calculate the mechanical anisotropy. The mechanical properties are critical in order to understand the energy dissipation mechanisms and hot spot formation of energetic materials, especially under extreme conditions; therefore, the [010] and [001] orientations with large shear stresses have a remarkable sensitivity.

#### 2.3.2. Simulated Raman Spectra of LLM-105 under Uniaxial Compressions 

**Figure 6:**
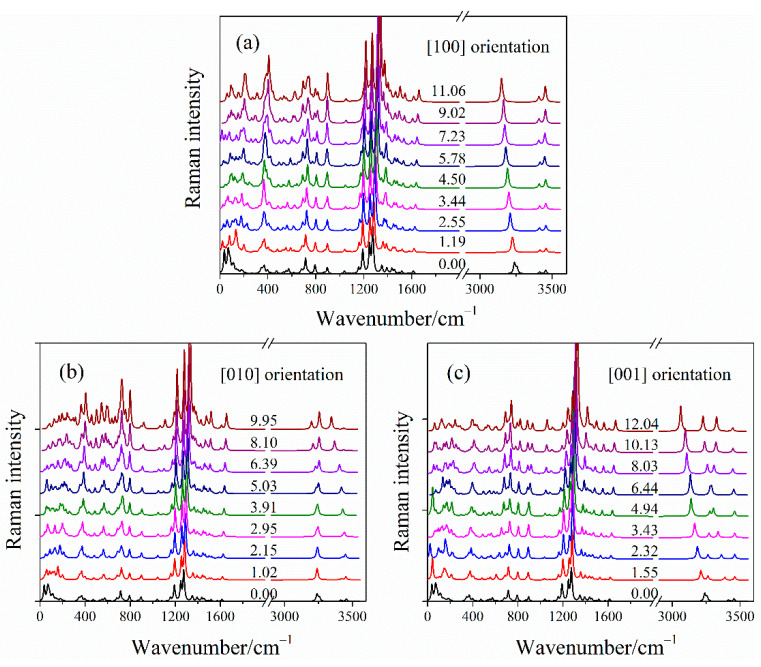
Simulated Raman spectra of LLM-105 along [100] (**a**), [010] (**b**), and [001] (**c**) orientations. The corresponding average principle stress is labeled on each curve.

**Figure 7:**
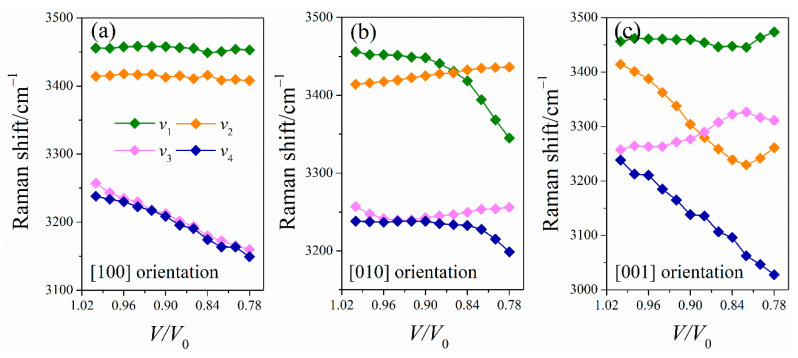
Raman shift of LLM-105 along [100] (**a**), [010] (**b**), and [001] (**c**) orientations.

The detailed knowledge of the vibrational properties of EMs under various loading conditions is necessary to understand the high-pressure behavior and anisotropy of LLM-105. Thus, the Raman spectra of LLM-105 under uniaxial conditions are calculated. In Figure 6, the pressure dependence of Raman spectra under uniaxial compressions is shown. The Raman spectra undergo considerable changes in the high-wavenumber range in [100] and [001] orientations. Detailed variations in the selected Raman shift of high-wavenumber modes are presented in Figure 7. 

Most vibrational frequencies shift gradually toward higher frequencies (i.e., blue-shift) with increasing pressure due to reduced interatomic distances. This behavior is consistent with the previous, high-pressure Raman experiment of the LLM-105 crystal [20]. The observed blue-shift in most vibrational frequencies is caused by the strengthening of chemical bonds. The inter- and intramolecular hydrogen bonds are enhanced under compression. Several vibrational modes show red-shift for three uniaxial compressions. The discontinuity of the Raman shift is observed along the [001] compression, which indicates the onset of possible phase transitions under uniaxial compressions. To understand such behavior, we examined the changes in the amino group of LLM-105, since it could provide insight into the stability of this crystal at extreme conditions in Figure 7. 

For the [100] orientation, the vibrational frequencies of the *v*_1_ and *v*_2_ modes are almost identical in the studied pressure range, which is similar to the case under hydrostatic conditions. The chemical bonds (N_1_H_1_ and N_2_H_3_) related to the *v*_1_ and *v*_2_ modes are insensitive at 0–10 GPa in Figure 7a. The compressibility values of N_1_H_1_ and N_2_H_3_ bonds are only 0.29% and 0.19%, respectively. By contrast, the N_1_H_2_ and N_2_H_4_ bonds lengthen with the increased pressure and are the main cause of the red-shifts of high-wavenumber modes (*v*_3_ and *v*_4_). Similarly, for [010] orientation, the *v*_1_ mode shows a remarkable red-shift, which is due to the lengthening of N_1_H_1_ covalent bonds. 

In addition, coupling modes are observed in the studied pressure range, which can be related to the intramolecular vibrational redistribution of Ems [28]. In Figure 7b, internal modes *v*_3_ and *v*_4_ have the same *A_g_* symmetry and vibrational patterns (NH_2_ symmetrical stretching). The initially low-intensity *ν*_3_ mode is enhanced and shifts with the increasing pressure to a higher frequency. Above *V/V*_0_ = 0.92, the intensity exchange and the avoided frequency crossing between the two modes are clearly observed. Such coupling mode contributes to the intramolecular vibrational energy transfer by the intensity exchange and avoided frequency crossing between different modes. 

For the [001] orientation, all NH bonds show discontinuous changes at *V*/*V*_0_ of 0.82. The shortening lengths of the N_1_–H_2_ bonds result in the blue-shift of the *v*_3_ vibrational mode, whereas the lengthening of other N–H covalent bonds can be treated as the main reason that leads to the red-shifts of high-wavenumber modes.

Overall, the vibrational properties of LLM-105 under hydrostatic and uniaxial conditions are simulated to examine its pressure effects and anisotropy. Based these results, the intermolecular hydrogen-bonded networks shrink and/or rearrange when the lattice space is rapidly compressed, which results in the redistribution of the electronic density at the atoms. As a result, the intramolecular geometry and the intermolecular interactions of LLM-105 are further modified and presented as distinct anisotropic responses.

## 3. Computational Methods

The first-principle calculations based on DFT were performed using the Cambridge Sequential Total Energy Package [29]. The exchange–correlation interaction was treated within the GGA–PBE function [30]. The semiempirical dispersion correction by Grimme (DFT-D2) [31] was used to account for the intermolecular noncovalent interactions of LLM-105 crystals. The Brillouin zone sample of Monkhorst–Pack [32] *k* grid of 0.05 Å^−1^ was adopted, and a kinetic energy cutoff of 1000 eV for the plane wave basis was used. The periodic boundary conditions of LLM-105 was employed and the unit cell parameters and atomic coordinates were fully relaxed by the Broyden, Fletcher, Goldfrab, and Shannon algorithms to obtain the equilibrium crystal structure of LLM-105 [33]. Among them, the convergence criteria of the maximum total energy of 5 × 10^−6^ eV per atom, with a maximum stress of 0.02 GPa, maximum force of 0.01 eV·Å^−1^, and maximum displacement of 5 × 10^−4^ Å were adopted for all calculations. Hydrostatic compressions were applied from 0 GPa to 10 GPa at a step of 0.5 GPa. Uniaxial compression was applied up to 78% of the equilibrium cell volume in steps of 2% along lattice directions. At each compression step, only atomic coordinates were allowed to relax, and the lattice was fixed. The maximum stress under uniaxial compression was less than 16 GPa in each lattice direction for improved contrast with hydrostatic compression.

On the basis of the optimized structure, the Raman spectrum was obtained within the density functional perturbation theory [34]. The wavenumbers of vibrational modes were obtained from the diagonalizable dynamical matrix. The Raman intensity was calculated using the linear response formalism combined with the dispersion correction method. The experimental factors of temperature (298 K) and incident light wavelength (514.5 nm) were considered for real Raman intensity. Then, the spectrum band was broadened by the Lorentzian function with 15 cm^−1^ width.

## 4. Conclusions

The pressure effects and anisotropic responses on the structure and vibrational properties of LLM-105 were systematically studied under hydrostatic and uniaxial compressions. Under hydrostatic pressure, the evolution of the lattice parameters of LLM-105 shows significant anisotropy, especially for the high compressibility in the *b*-axis. Furthermore, the red-shifts of several vibrational modes are obtained under hydrostatic pressure due to the enhanced hydrogen bonds resulting from a reduced intermolecular distance. Based on the diagonal elements of the stress tensor, the shear stresses of LLM-105 under uniaxial compressions are calculated and suggest that LLM-105 can be more sensitive along the [001] orientation than the [100] and [010] orientations. Furthermore, the Raman spectra of LLM-105 crystal under uniaxial compressions are simulated and display a remarkable anisotropy. The discontinuity of the Raman shift is observed along the [001] orientation, which indicates the onset of possible phase transitions under uniaxial compressions. The coupling vibrational modes, which are observed along [010] compressions, are related to the intramolecular vibrational redistribution. This study provides a deep understanding of the high-pressure behavior and anisotropy of LLM-105 under different pressure loadings.

## Figures and Tables

**Table 1:** Calculated and experimental lattice parameters and unit cell volume of LLM-105 at ambient pressure.

	*V/*Å [3]	*a/*Å	*b/*Å	*c/*Å	*β/*°
Expt. [5]	748.16	5.716	15.850	8.414	101.041
Expt. [9]	747.3	5.72	15.82	8.42	101.15
Expt. [10]	750.08	5.723	15.870	8.424	-
GGA/PW91 [13]	939.23	6.008	18.279	8.706	100.75
LDA/CA-PZ [13]	737.24	5.837	15.844	8.416	99.51
PBE (HASEM) [15]	747.38	5.64	15.96	8.46	100.93
Force field [18]	711.00	5.64	15.55	8.24	100.96
This work	756.09	5.758	15.750	8.500	101.238
Deviation/%	1.06	0.73	−0.63	1.02	0.19

**Table 2:** Calculated and experimental bulk modulus of LLM-105 at ambient pressure.

	*B*_0_ (GPa)	*B*_0_^′^ (GPa)
This work	16.03	8.67
Expt. [9]	11.19	18.54
Expt. [10]	15	9
Expt. [11]	19.23	6.70
Calc. [15]	16.5	9.4
Calc. [18]	13.8	11.7

## Data Availability

The data presented in this study are available from the corresponding author.

## Supplementary Materials

The following are available online, Table S1: Characteristics of vibrational modes in LLM-105 crystal at ambient pressure. *dv*/*dp* is slope of pressure-induced Raman shift. Abbreviation: st: stretch, sci: scissor, tw: twist, bre: breathe, def: deformation, sym: symmetric, asym: asymmetric; Figure S1: The evolutions of NH_2_ bond length and angle of LLM-105 molecular under hydrostatic pressure; Figure S2: Three principal stresses σ_xx_, σ_yy_ and σ_zz_ as function of compression ratio *V*/*V*_0_ under uniaxial loading; Figure S3: The average principal stress σ as function of compression ratio *V*/*V*_0_ under different uniaxial loading.

## Sample Availability

Samples of the compounds are available from the authors.
