# Supplementary material for: Compression Behavior and Vibrational Properties of New Energetic Material LLM-105 Analyzed Using the Dispersion-Corrected Density Functional Theory
Source: Molecules. 2021 Nov 12;26(22):6831. doi: 10.3390/molecules26226831 (PMC8625217; doi:10.3390/molecules26226831)
Supplement: Supplementary file 1 [file molecules-26-06831-s001.zip › molecules-1404870-supplementary.pdf]

Supporting Information

Compression behavior and vibrational properties of new energetic material LLM-105 analyzed using the dispersion-corrected density functional theory

Tianming Li,[2] Junyu Fan,[1,2,3,*] Zhuoran Wang,[2] Hanhan Qi,[2] Yan Su,[2,*] Jijun Zhao[2]

[1]*Department of Physics, Taiyuan Normal University, Taiyuan 030619, China*

[2]*Key Laboratory of Materials Modification by Laser, Ion and Electron Beams (Dalian University of Technology), Ministry of Education, Dalian 116024, China*

[3]*Institute of Computational and Applied Physics, Taiyuan Normal University, Jinzhong 030619, China*

[*] Corresponding author. E-mail: su.yan@dlut.edu.cn, fanjunyu@tynu.edu.cn

Table S1. Characteristics of vibrational modes in LLM-105 crystal at ambient pressure. dv/dp is slope of pressure-induced Raman shift. Abbreviation: st: stretch, sci: scissor, tw: twist, bre: breathe, def: deformation, sym: symmetric, asym: asymmetric.

Mode	Wavenumber/cm^{-1}			Assignments	dv/dp/cm^{-1} GPa^{-1}	
	A_g	B_g	Expt.[S1]		This work	Expt. [S1]
v_1	3456	3456	3439	NH_2 asym. st.	-0.31	
v_2	3414	3416	3403	NH_2 asym. st.	-2.44	
v_3	3257	3257	3300	NH_2 sym. st.	-6.33	
v_4	3238	3245	3281	NH_2 sym. st.	-6.13	
v_5	1614	1620	1650	NH_2 sci., C-NH_2 st.	4.40	5.04
v_6	1575	1577	1643	NH_2 sci., C-NH_2 st.	3.53	4.86
v_7	1518	1509	1600	NH_2 sci.	3.39	3.96
v_8	1495	1495	1566	NH_2 sci.	3.17	3.42
v_9	1455	1459	1551	NH_2 sci., ring def.	4.24	2.70
v_{10}	1436	1437	1519	NO_2 st., NH_2 sci., ring def.	3.23	3.78
v_{11}	1409	1432	1499	NH_2 sci., NO_2 st.	3.02	3.96
v_{12}	1393	1408	1474	NH_2 sci., NO_2 st.	1.26	3.96
v_{13}	1350	1354	1456	HN_2 rock, ring st.	4.86	2.52
v_{14}	1333	1337		HN_2 sci., ring st.	3.79	
v_{15}	1275	1281	1378	HN_2 rock, ring bre., NO_2 st.	5.41	5.94
v_{16}	1249	1249	1265	HN_2 sci., ring bre., NO_2 st.	3.13	3.06
v_{17}	1192	1204	1251	HN_2 sci., ring def., NO_2 st.	2.69	2.34
v_{18}	1158	1167	1188	NH_2 rock, N-O rock	3.06	3.42
v_{19}	1078	1081	1089	HN_2 rock, ring bre.	2.58	3.60
v_{20}	1039	1040	1062	NH_2 rock, ring st.	1.49	2.34
v_{21}	896	898	921	NH_2 rock, ring st.	1.26	1.71
v_{22}	869	866	893	NH_2 rock, ring st., NO_2 st.	3.02	
v_{23}	794	791	817	NH_2 rock, ring st., NO_2 st.	1.46	1.59
v_{24}	739	739	759	NH_2 rock, ring def., NO_2 st.	3.93	1.10
v_{25}	732	732	736	NH_2 tw., ring def., NO_2 st.	1.30	2.45
v_{26}	715	716	715	NH_2 st., ring st.	2.66	1.35
v_{27}	709	713	703	NH_2 tw.	1.91	1.84
v_{28}	696	697		NH_2 rock, ring def., NO_2 tw.	2.25	
v_{29}	686	691		NH_2 tw.	1.84	
v_{30}	681	681		NH_2 tw.	1.89	
v_{31}	673	674	637	NH_2 tw., ring def., NO_2 tw.	0.09	0.98
v_{32}	617	617		NH_2 tw.	2.97	
v_{33}	574	576	560	NH_2 wag	5.34	3.92
v_{34}	552	552	544	NH_2 wag	5.30	0.98
v_{35}	531	533		NH_2 tw., ring def.	1.63	
v_{36}	530	531		NH_2 wag	0.80	
v_{37}	472	476	485	NH_2 tw., ring def., NO_2 tw.	3.63	3.92
v_{38}	426	426	434	NH_2 tw., ring def., NO_2 tw.	1.13	1.75

v_{39}	396	401	415	HN_2 rock	1.85	2.96
v_{40}	370	372	379	HN_2 rock	3.13	2.85
v_{41}	361	360	354	HN_2 rock, NO_2 sci.	2.48	3.95
v_{42}	349	346	344	NH_2 st., ring def., NO_2 st.	3.14	3.07
v_{43}	342	339	336	NH_2 tw., ring def., NO_2 tw.	3.41	
v_{44}	332	329	272	NH_2 tw., ring def., NO_2 tw.	3.17	
v_{45}	269	275	267	NH_2 wag, ring def.	2.35	2.74
v_{46}	191	210	196	NH_2 tw., ring def., NO_2 rock	6.41	
v_{47}	160	180	179	lattice vibration	9.24	6.36
v_{48}	157	151	172	lattice vibration	7.78	
v_{49}	139	130	145	NH_2 tw., ring def., NO_2 tw.	8.20	6.47
v_{50}	117	126		NO_2 rock	8.36	
v_{51}	106	111	110	lattice vibration	7.51	7.78
v_{52}	103	107	100	lattice vibration	6.89	
v_{53}	84	91	88	lattice vibration	7.69	6.69
v_{54}	77	86	76	lattice vibration	4.06	7.13
v_{55}	70	72	60	NH_2 rock, ring rock, NO_2 rock	3.76	4.60
v_{56}	61	59	51	lattice vibration	1.34	4.28
v_{57}	37	29		lattice vibration	2.20	

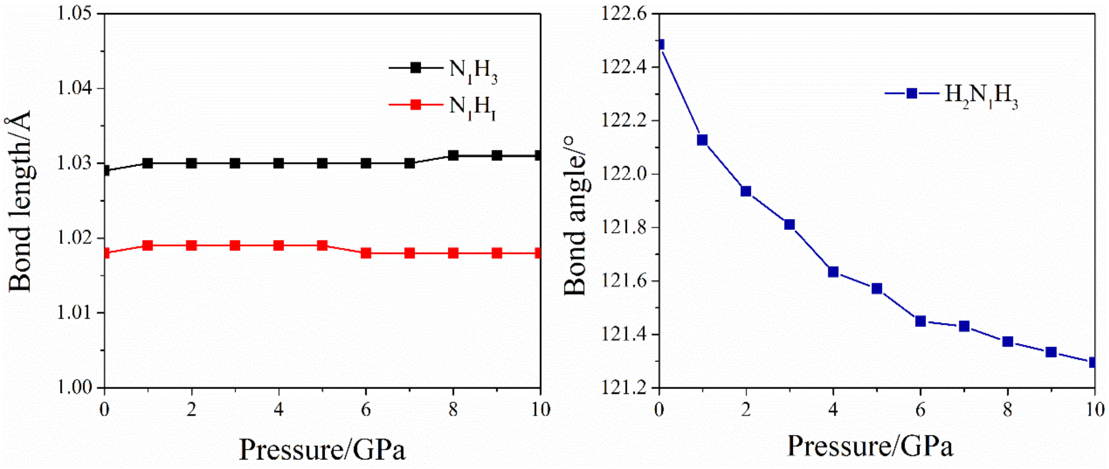

Figure S1. The evolutions of NH_2 bond length and angle of LLM-105 molecular under hydrostatic pressure.

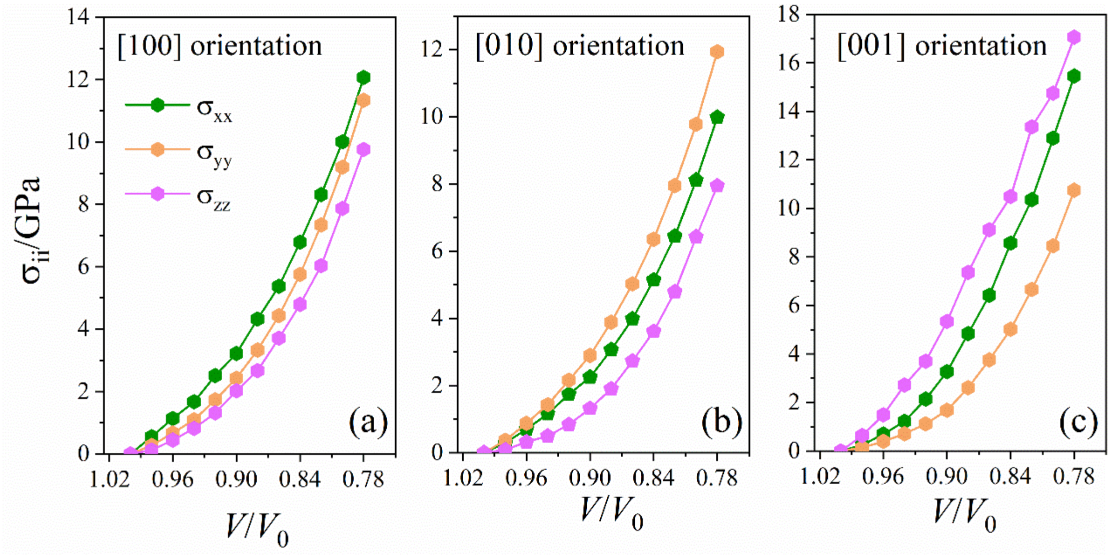

Figure S2. Three principal stresses σ_{xx}, σ_{yy} and σ_{zz} as function of compression ratio V/V_0 under uniaxial loading.

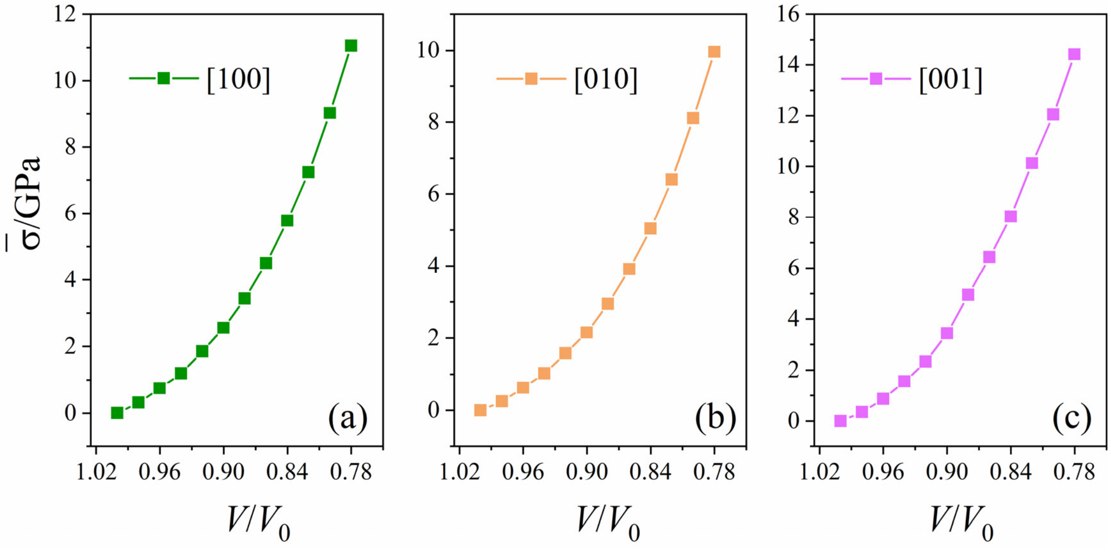

Figure S3. The average principal stress $\overline{\sigma}$ as function of compression ratio V/V_0 under different uniaxial loading.

References

S1. Xu, Z., et al., Pressure- and Temperature-Dependent Structural Stability of LLM-105 Crystal. *The Journal of Physical Chemistry C* **2018**, *123*, 1110-1119.
